# The association between preschoolers’ retinal microcirculation and the indoor microbial environment: results of the ENVIRONAGE birth cohort

**DOI:** 10.1038/s41598-025-18379-y

**Published:** 2025-10-06

**Authors:** Wouter van Dyck, Martin Täubel, Pauli Tuoresmäki, Yinthe Dockx, Leen Luyten, Leen Rasking, Patrick De Boever, Tim S. Nawrot, Lidia Casas

**Affiliations:** 1 Department of Family Medicine and Population Health, Social Epidemiology and Health Policy (SEHPO), University of Antwerp, Universiteitsplein 1, Wilrijk, 2610 Belgium; 2 Finnish Institute for Health and Welfare, Environmental Health Team, Neulaniementie 4, Kuopio, 70701 Finland; 3 Centre for Environmental Sciences, Hasselt University, Agoralaan, Diepenbeek, 3590 Belgium; 4 Antwerp University Hospital (UZA), Drie Eikenstraat 655, Edegem, 2650 Belgium; 5 Department of Public Health, Center for Environment and Health, KU Leuven, Herestraat 49, Leuven, 3000 Belgium; 6 Institute for Environment and Sustainable Development (IMDO), University of Antwerp, Universiteitsplein 1, Wilrijk, 2610 Belgium; 7 Laboratory of Applied Microbiology and Biotechnology (LAMB), Department of Bioscience Engineering, University of Antwerp, Groenenborgerlaan 171, Antwerp, 2020 Belgium

**Keywords:** Indoor microbial environment, Retina microcirculation, Pre-school age children, Epidemiology, Microbiology

## Abstract

Early life environmental microbiota may influence normative development. Here, we explore the associations between the residential indoor microbial environment and the retinal microcirculation among preschoolers. We included 177 children aged 4–6 years from the Belgian ENVIRONAGE birth cohort. We measured retina microcirculation using fundus photography and quantified the retinal vessel tortuosity [tortuosity index (TI)] and diameters [central retinal vein equivalent (CRVE) and central retinal artery equivalent (CRAE)]. Residential indoor microbial characteristics (bacterial and fungal loads, richness, diversity, and taxa) were measured in settled dust using qPCR and amplicon sequencing. Adjusted associations were obtained using linear regression models and expressed as coefficients (β) with 95% confidence intervals (CI). We observed inverse associations between microbial loads and retinal microcirculation, significant for CRAE: β = -0.28; CI:-0.53;-0.04 (bacteria) and β = -0.27; CI:-0.50,-0.03 (fungi). Conversely, retinal microcirculation was directly associated with Gram-positive bacterial loads, significant for TI (β = 0.44; CI:0.06,0.81). These associations were stronger among boys. No consistent associations were observed for diversity. Conclusively, indoor microbial loads can affect the retinal microcirculation in preschool children. Retinal vascularization is a cardiovascular marker linked to immune factors and brain vascularization. Our findings support previously observed associations of the environmental microbiome with cognition, and open new hypotheses about potential cardiovascular effects.

**Supplementary Information:**

The online version contains supplementary material available at 10.1038/s41598-025-18379-y.

## Introduction

Children spend most of their time indoors, according to an American behavior survey^1^. There, they are exposed to indoor environmental factors like the indoor microbiome. Exposure to an imbalanced environmental microbiome has been hypothesized to impact the human microbiome and immune system’s development^2–4^, resulting in a variety of health outcomes in the gut^5^, the lung^6^, the immune system^7–9^, and the brain^10,11^. Early life is a critical exposure time window during which environmental factors can induce long-lasting health consequences^12^. Indeed, results from previous research suggest that early life exposure to a quantitatively rich and diverse (many different species approaching an even distribution) microbiome may protect against the development asthma, atopy, and allergies^7,8,13–18^. In addition, these early life exposures were also suggested to impact cognitive development and behavior^10,11,19–21^. Since the underlying mechanisms for these associations are hypothesized to be immunological, there may be other health effects that have not yet been explored.

The retinal microcirculation, often characterized by retinal vessel diameter metrics, is an integral part of the cardiovascular system that is affected by environmental factors like air pollution^22–24^. Changes in retinal vessel diameters have been linked to multiple immunological health outcomes like rheumatism, acute infections and inflammation recovery^25–31^. The retina is an outgrowth of the brain and the retinal microcirculation is also considered a proxy for brain microcirculation^32,33^, making it an accessible biomarker for assessing the cerebrovascular status. Wider retinal venules would result in a sub-optimal oxygen supply to the brain^34,35^, and this has been associated with decreased cognitive functions^19,36^, increased risk for dementia^37^, cerebrovascular diseases^38,39^ and schizophrenia^40,41^. Here, we hypothesize that an early life exposure to a rich and diverse environmental microbiota in the residence can impact the retinal microcirculation. These hypothesized vascularization changes could explain, among others, the observed brain effects of exposures to a rich and diverse indoor microbial environment, and open new hypothesis regarding other potential beneficial effects of early life microbial exposures. In this study, we aim to explore the associations of the residential bacterial and fungal indoor microbiome diversity, load and composition with metrics of the retinal microcirculation in children aged 4 to 6 years, and assess the potential effect modification of sex in these associations.

## Materials and methods

### Study design and population

We use data collected in the ENVIRONAGE project^42^. This Belgian birth cohort of mother-child pairs started in 2010 with the objective of studying the impact of several exposures on aging. Since 2010, mother-newborn pairs are being recruited at the East-Limburg Hospital in Genk. Pregnant women with a planned cesarian section and non-Dutch speaking women were excluded. To date, more than 2500 mother-child pairs have been included and are followed in the life-course study. One contact moment is at the child’s age of four to six years when assessments are performed. The assessment includes the administration of questionnaires on socio-demographics, residential address, health and lifestyle factors to the mothers, and clinical exams of the children, including fundus pictures and blood pressure measurements. In 2017 and 2018, participants attending this four-to-six-year follow-up were invited to join a microbiome sub-study in which settled dust was collected at their homes, and additional questionnaires including information on household characteristics were administered. In total, 284 children were eligible to participate in the sub-study and we were able to contact 233 of those. From the contacted families, 189 accepted to participate in the microbiome sub-study. Dust collection was not performed in eight households due to logistical reasons. From the 181 households where dust was collected, two were excluded due to insufficient amounts of dust and one because the duration of sampling exceeded the maximum of nine weeks. From the resulting 178 household samples, two reported irregularities during sampling. Lastly, one household had two siblings that were both included in the analysis. As a result, this study includes information of 176 households and 177 children.

The study protocol was approved by the ethical committee of Hasselt University and complied with the Helsinki Declaration. Parents gave written informed consent and children verbal permission (reference number B9115201836553).

### Retinal microcirculation

Fundus pictures of both eyes from all children were taken with a 45° 6.3-megapixel digital nonmydriatic retinal camera (Canon CR-2 plus; Hospithera NV). The dimensions (diameter, width, length and distances) of the optic disc, largest arterioles and venules were calculated using the MONA-REVA software, version 2.1.1 (VITO Health). The retinal images were analyzed using MONA REVA vessel analysis software (version 2.1.1) developed by VITO (Mol, Belgium; https://vito.be/nl). Central Retinal Arteriolar Equivalent (CRAE) and Central Retinal Venular Width Equivalent (CRVE) were determined from the retinal images as proxies for retinal blood vessel widths. The following procedure was used to obtain CRAE and CRVE metrics. Consistent retinal regions were obtained across all the fundus images in MONA REVA by defining an annular region centered on the optic disc, with the inner and outer radii of the annulus set at 1.0 and 3.0 times the radius of the optic disc, respectively. Next, the image analysis algorithm based on a multiscale line filtering algorithm automatically segmented the retinal vessels. Post-processing steps such as double thresholding, blob extraction, removal of small connected regions, and filling holes were performed. The diameters of the retinal arterioles and venules that passed entirely through the circumferential zone 0.5 to 1 disc diameter from the optic disc margin were calculated automatically^43^. A trained grader verified and corrected vessel diameters and vessel labels (arteriole or venule) with the MONA REVA vessel editing toolbox. The diameters of the 6 largest arterioles and 6 largest venules were used in the revised Parr-Hubbard formula for calculating the Central Retinal Artery Equivalent (CRAE) and Central Retinal Venular Equivalent (CRVE)^44^. The CRAE and CRVE values of both images were averaged and used in the statistical analysis. A measure of the vessel path, i.e. the tortuosity index (TI), calculated as the mean tortuosity (distance of the actual path of a branch segment - Euclidean distance from start to end / distance of the actual path of a branch segment) within the zone 0.5 until 2 times the optic disc diameter. The tortuosity index was multiplied by 100 to represent the percentage tortuosity.

### Indoor environmental microbiota

All indoor microbial measurements were performed from settled dust samples collected during spring (to control for the impact of season on indoor microbiota) in the living room of the participants, as previously detailed in Dockx et al. 2021^45^. Sterile petri dishes (92 × 16 mm) were positioned open approximately 2m above the ground and away from any major air flows in the living rooms for four to nine weeks. After the sampling period, petri dishes were closed, sealed and stored at -20 °C until further processing. The processing included collection of the settled dust in the petri dishes with a cotton swab and subsequent DNA extraction from the dust. The cotton swab tip was wettened in sterile water with 0.05% Tween 20 solution before thoroughly swabbing the bottom and then the lid of the petri dish. After swabbing, the tip of the cotton swab was cut and placed into a DNA extraction tube containing glass beads. The tubes were stored at -20 °C for DNA extraction and sequencing. The frozen DNA extraction tubes were shipped on dry ice to the Finnish Institute for Health and Welfare (Kuopio, Finland). DNA was extracted from the dust swabs via mechanical cell disruption in a bead-milling step followed by extraction and clean-up using Chemagic DNA Plant kit (PerkinElmer chemagen Technology GmbH, Germany). Samples were sent on dry ice to the sequencing service partner LGC Genomics (Germany). Details on the DNA extraction, bacterial and fungal 16 S and ITS PCR amplification and amplicon sequencing, as well as the sequence processing in this study have been provided in Dockx et al. 2021^45^. In brief, the V4 region of the bacterial 16 S rRNA gene and the ITS1 region of the fungal Internal Transcribed Spacer (ITS) were amplified using primer pairs 515 F/806R^46^ and ITS1F/ITS2^47^, respectively. Sequencing was performed on an Illumina MiSeq with V3 chemistry resulting in paired-end reads with a length of 300 bp each. The bacterial and fungal amplicon data were analyzed using standard dada2 pipeline version 1.8^48^. Taxonomy was assigned using SILVA^33^ database version 132 for bacteria and UNITE database version 7.2 for fungi^49^. Downstream processing included removal of chimeras, chloroplast, and mitochondria sequences, as well as of potential contaminants utilizing negative controls and Decontam package version 1.2^50^. QIIME software version 1.9.1^51^ was used to calculate Chao1 richness and Shannon diversity indices. Fungal load, Gram-positive bacterial and Gram-negative bacterial loads in house dust were determined using quantitative PCR (qPCR) method and previously described assays^52^. Number of microbial cell equivalents (CE) in the sample was calculated using relative quantification^53^ and the microbial loads in settled dust were normalized for sampling surface area and sample accumulation duration, and were expressed as CE per m^2^ of settling surface per day. In this study, the Shannon diversity Index (a measure taking into consideration both the richness and evenness of species in the sample), Chao 1 richness index (an estimate of true species richness, without taking evenness into account), the total bacterial and fungal loads (expressed as CE per m^2^ of settling surface per day) and the percentage of Gram-positive and Gram-negative bacterial loads (calculated by dividing the Gram-positive and Gram-negative bacterial load, respectively, by the total bacterial load and expressed as percentages), were selected as the main exposures of interest based on previous research^11,45,54^.

### Potential confounders, modifiers and other covariates

We considered socio-economic status, air pollution and the proportion of residential green as potential confounders (see Fig. S1: DAG in the appendices)^45,54,55^. For socio-economic status of the participant, we used maternal education. This information was extracted from the questionnaires administered to the mother at follow-up. Maternal education was captured as a categorical variable with 3 levels: “low” (no diploma or primary school), “middle” (high school), or “high” (college or university degree). Because of the low number of participants with mothers reporting primary school or less (*n* = 6), the low and middle categories were grouped into one category.

Data on air pollution was collected by geocoding the residential address and assigning an estimation of the yearly average concentrations of common traffic-related air pollutants at the time of microbiome measurement at the residential address and the proportion of green spaces surrounding the residence. In this study, we used black carbon concentrations as measure of air pollution because it was used previously^45^ and, in sensitivity analyses, we evaluated this choice by switching the measure to NO2 concentrations. The average concentrations of black carbon and NO2 at the residential address were provided by IRCELINE (http://www.irceline.be/). They use data from monitoring stations and a spatiotemporal interpolation method^56^ to predict the concentrations of air pollutants at each coordinate in Belgium. The details of these methods are described elsewhere^42^.

Data on residential green was represented by the percentage of high outdoor green within 50 m of the residence. This was estimated by using data from the Green Map of Flanders (GF) 2012 (Agency for Geographic Information Flanders, AGIV) in the Geographic Information System (GIS) ArcGIS 10 software. Total green included all non-agricultural vegetation and was divided in low and high green with a cut-off of 3 m high. We selected the proportion of high green (vegetation higher than 3 m) as a proxy for residential green because this type of residential green can shape the characteristics of the indoor environmental microbiome^45^. We evaluated this choice by replacing high green with low green in the sensitivity analysis.

In addition to the selected potential confounders, we considered known important determinants of retinal vascularization like child’s BMI and mean arterial blood pressure. Both of these were determined at follow-up by weighing the child and measuring blood pressure with an automated upper-arm blood pressure monitor (Omron Corporation). The device was equipped with a cuff adapted to the arm size of children. The standardized methods, as described by the European Society of Hypertension, were used. After 5 measurements, MAP was calculated as the average diastolic blood pressure plus one-third of the average pulse pressure (the difference between systolic- and diastolic blood pressure). More details on these blood pressure measurements are described by Martens et al.^57^. Next to determinants of the retinal vascularization, the microbiome duration of sampling was taken into account (defined as the number of days that the petri dish was passively collecting dust in the home’ living room). Lastly, we considered the sex of the child as potential effect modifier because CRAE and CRVE was significantly different in boys and in girls in our dataset. In addition, research shows that boys play more outside^58,59^ and could therefore be exposed differently as girls.

### Statistical analysis

All data was pooled, cleaned and analyzed using R statistical software version 4.2.2. There were 17 children with missing retinal pictures (9.6%). There was no significant difference for any of the exposures or covariates between the populations with and without missing outcome data (descriptives presented in Table S1 in the supplementary materials). Therefore, in the main analyses we assumed that these were missing completely at random (MCAR) . In addition, the air pollution concentrations were not available for 2 households and the age at follow-up was unknown for 4 children. Due to the low percentage of missingness and the assumption of MCAR, we opted for a complete case analysis. However, in the sensitivity analysis, a multiple imputation with 3 iterations was performed to evaluate the assumption and impact of missingness on the models. All missing variables were imputed by classification and regression trees. These data were pooled to build new models.

Univariate relations within our data were explored to place the gathered data in context. We built linear regression models for the multivariate analysis, adjusting for the abovementioned confounders (maternal education, black carbon concentrations and residential high green proportions), retinal microcirculation related variables (mean arterial blood pressure and BMI) and duration of dust sampling. In the main analyses, we used separate models for Chao1 index, Shannon index and microbial loads. For modeling, the Shannon and Chao1 indices together with the fraction of Gram-positive bacterial load (expressed in percentages) were standardized over their interquartile range (IQR). Microbial loads were log-transformed with a natural logarithm to comply with the linear regression model assumptions. These models were built on the complete data set and stratified by sex. Associations are expressed as regression coefficients (β) and their 95% confidence intervals (CI) per IQR increments for Shannon and Chao1 indices. For the bacterial and fungal loads, the regression coefficient was back transformed (β*log(1.1)), and the results are expressed as percentage change per 10% increments for microbial loads.

To study whether the results were carried by the microbiota summary variables (diversity, richness and total loads) or by a few relevant genera, ANCOM BC2^60^ and random forest methods were used to identify which bacterial and fungal genera are most contributing to the retinal microcirculation characteristics. ANCOM BC2 was performed on bacterial and fungal genera separately. All genera with a q value below 1 were used in further analysis. Random forests were also built for bacterial and fungal genera separately. The top 5 most important nodes in the forest were used in further analysis. The list of relevant genera was described and used as adjustment variables in the linear models of the main analysis to see their influence on the relation between the microbial summary statistics and the child’s retinal microcirculation. These adjustments were done in 4 different ways: using the relative abundance or absolute abundance (relative abundance * load) for both the total set of influential variables and the set of influential variables that have the same direction of association as the exposure does with the outcome (i.e. either increase or decrease in abundance by retinal microcirculation outcome measure).

To study whether the results were carried by the population or by a few observations, the effect of influential observations on the model results was assessed. Influential observations were defined as outcomes that had a Cook’s distance of more than 4 times the mean Cook’s distance. After these observations were defined, the models were fitted again while ignoring the influential observations.

In the sensitivity analyses, we evaluated the impact of the choice of proxy variables for blood pressure, BMI, air pollution and residential green on the associations by building models with substitute variables. Mean arterial blood pressure was replaced by diastolic blood pressure, BMI by zBMI (BMI relative to the median BMI for that sex and age group), black carbon was replaced by the residential NO2 concentrations and high residential green within 50 m of the house was replaced by low residential green within 50 m of the house. Lastly, as previously mentioned, the complete-case analysis approach was evaluated by performing the same analysis on a multiple imputed data set (3 iterations). This sensitivity analyses were conducted to address a potential for selection bias introduced by missing values. For all analysis, a significance level of 0.05 was used to evaluate statistical significance.

## Results

### Description of the study population

The characteristics of the study population are described in Table 1. From the 177 children, about half were girls. Except for mean arterial blood pressure, CRAE and CRVE, none of the participant-, maternal- or residential characteristics differed significantly between boys and girls. CRAE and CRVE were higher in girls compared to boys. At the time of retinal imaging, children had a median age of 4.4 years old (IQR = 0.4 years) and their mothers were approximately 30 years old. Over two thirds of the mothers had a high education. Approximately a third of the bacterial load in house dust was Gram-positive. The residences were sampled for a median of 42 days.

**Table 1:** Description (median and IQR) of the characteristics of the study population and residence environmental factors for all population and stratified by sex.

	Total (*n* = 177)	Girls (*n* = 88)	Boys (*n* = 89)
Child characteristics			
Age (years)	4.4 (0.4)	4.5 (0.5)	4.4 (0.3)
BMI (kg/m^2^)	15.9 (1.5)	16.0 (1.7)	15.9 (1.4)
Mean arterial BP* (mm Hg)	71.6 (7.3)	72.2 (7.4)	70.5 (6.9)
Retina microcirculation			
CRVE (µm)*	240 (28.3)	245 (30.2)	237 (24.0)
CRAE (µm)*	173 (18.8)	177 (17.2)	170 (22.5)
TI (%)	89.2 (2.3)	89.0 (2.3)	89.3 (2.1)
Maternal characteristics			
Age (years)	30.0 (5.0)	30.0 (5.0)	30.0 (5.0)
High education (n and %)	123 (69.5%)	61 (69.3%)	62 (69.7%)
Residential environment factors			
Bacteria in settled dust			
Shannon index	7.1 (1.1)	7.2 (1.0)	7.1 (1.1)
Chao1 index	406 (211)	411 (181)	397 (215)
Total Load (CE/m2/day)	500,000 (539,000)	454,000 (564,000)	442,000 (534,000)
Percentage Gram-positive Load (%)	37.1 (15.1)	37.3 (12.6)	36.6 (16.8)
Fungi in settled dust			
Shannon index	3.4 (1.3)	3.5 (1.3)	3.3 (1.3)
Chao1 index	130 (81.9)	130 (78.2)	130 (74.2)
Load (CE/m2/day)	35,000 (44,000)	33,000 (36,000)	36,000 (46,000)
Duration of dust sampling (days)	42.0 (6.0)	42.0 (6.0)	42.0 (6.0)
High greenness** within 50 m of the residence (%)	7.8 (15.1)	7.5 (12.9)	8.8 (16.6)
BC (µg/m3)	1.1 (0.2)	1.2 (0.2)	1.1 (0.2)

BMI: Body Mass Index; BP: blood pressure; CRVE: central retinal vein equivalent; CRAE: central retinal arterial equivalent, TI: tortuosity index, BC: black carbon.

*Significant difference between boys and girls.

**Vegetation > 3 m height.

### Correlations between retinal microcirculation and the indoor environmental microbiota

**Fig. 1:**
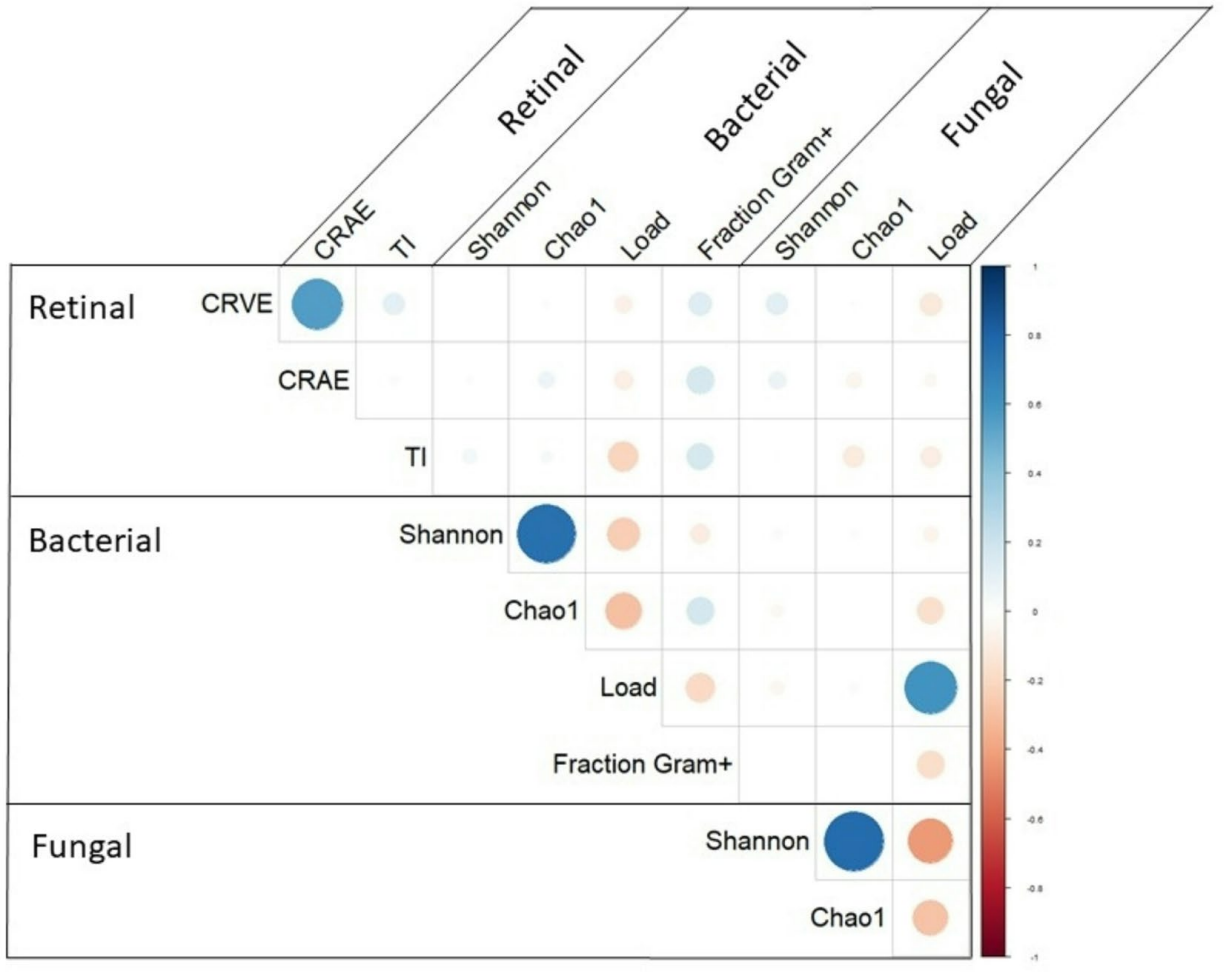
Correlation matrix of microbial exposures and retinal microcirculation characteristics in the complete study population. The size of the circle indicates the strength of the correlation. The color of the circle indicates the direction of the correlation with blue being directly correlated and red inversely. CRVE: central retinal vein equivalent; CRAE: central retinal arterial equivalent, TI: tortuosity index.

The correlations between the retinal microcirculation characteristics and the microbial exposures are depicted in Fig. 1. CRVE and CRAE were strongly and directly correlated to each other. Shannon and Chao1 alpha diversity indices were directly correlated to each other but inversely correlated to their respective loads. When comparing microbial exposures to retinal microcirculation outcomes, the total bacterial load was weakly, inversely correlated to TI with a correlation coefficient (r) of -0.21 (CI = -0.35 ; -0.06). In addition, the fraction of Gram-positive bacterial load was directly and weakly correlated to both TI (*r* = 0.17, CI = 0.01 ; 0.31) and CRAE (*r* = 0.17, CI = 0.01; 0.32). After stratifying by sex (Figure S2 in the supplementary materials), we observe stronger correlations among the boys compared to girls, but only significant between TI and the total bacterial load in boys (*r* = -0.34, CI = -0.52; -0.13). The correlations between exposure and outcome were weaker and never significant in girls. A figure showing the correlations between all variables included in this study can be found in the supplementary material (figure S3).

### Adjusted associations between retinal microcirculation and the indoor environmental microbiota

**Fig. 2:**
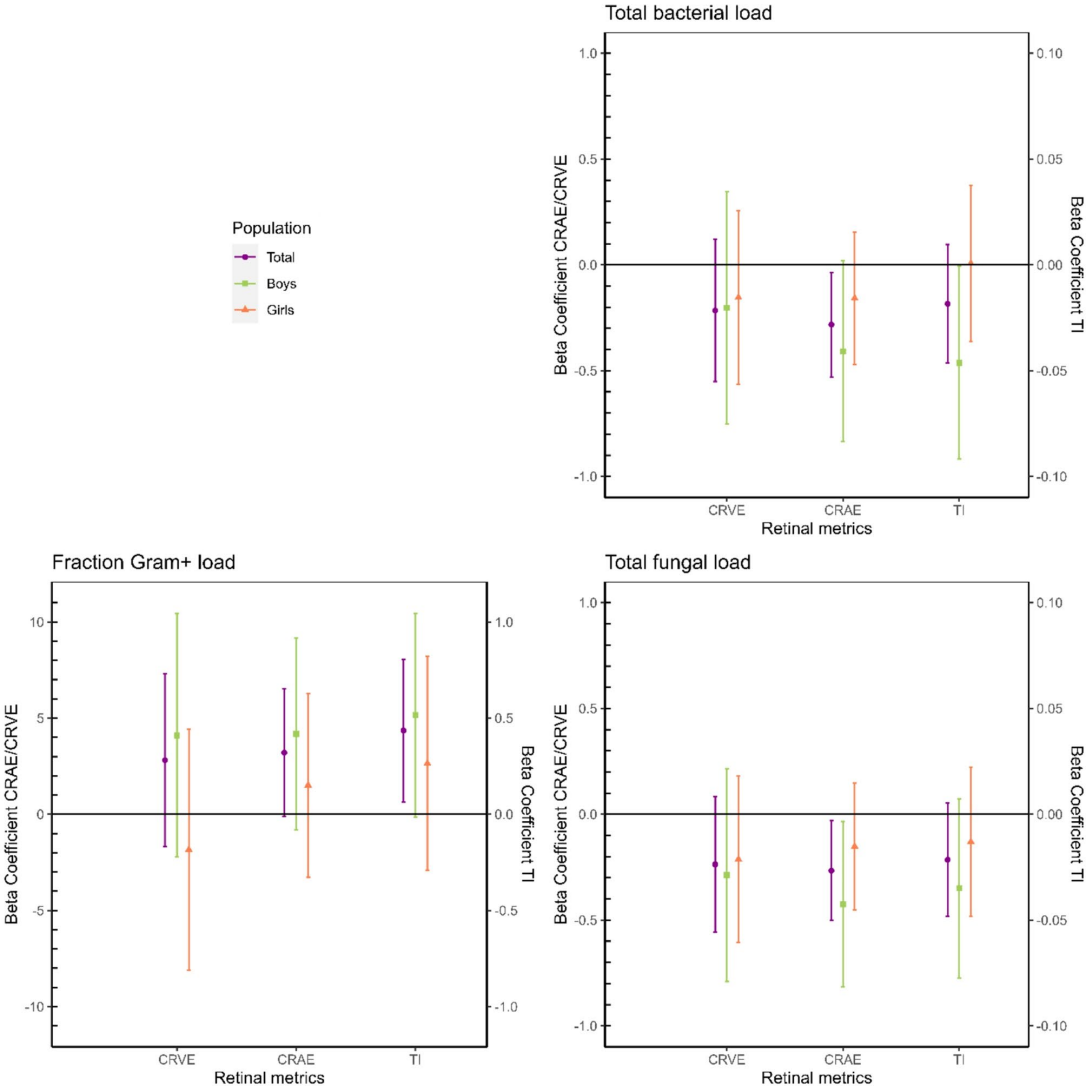
Adjusted beta coefficients and their 95% confidence intervals (CI) for microbial loads versus retinal microcirculation outcomes in the total population (*n* = 177, purple), boys (*n* = 89, green) and girls (*n* = 88, orange). Models were adjusted for exposure counterpart (bacteria/fungi), BMI, mean arterial blood pressure, education of the mother, black carbon concentrations, proportion of high green around the residence and settled dust duration of sampling. CRVE: central retinal vein equivalent; CRAE: central retinal arterial equivalent, TI: tortuosity index.

**Fig. 3:**
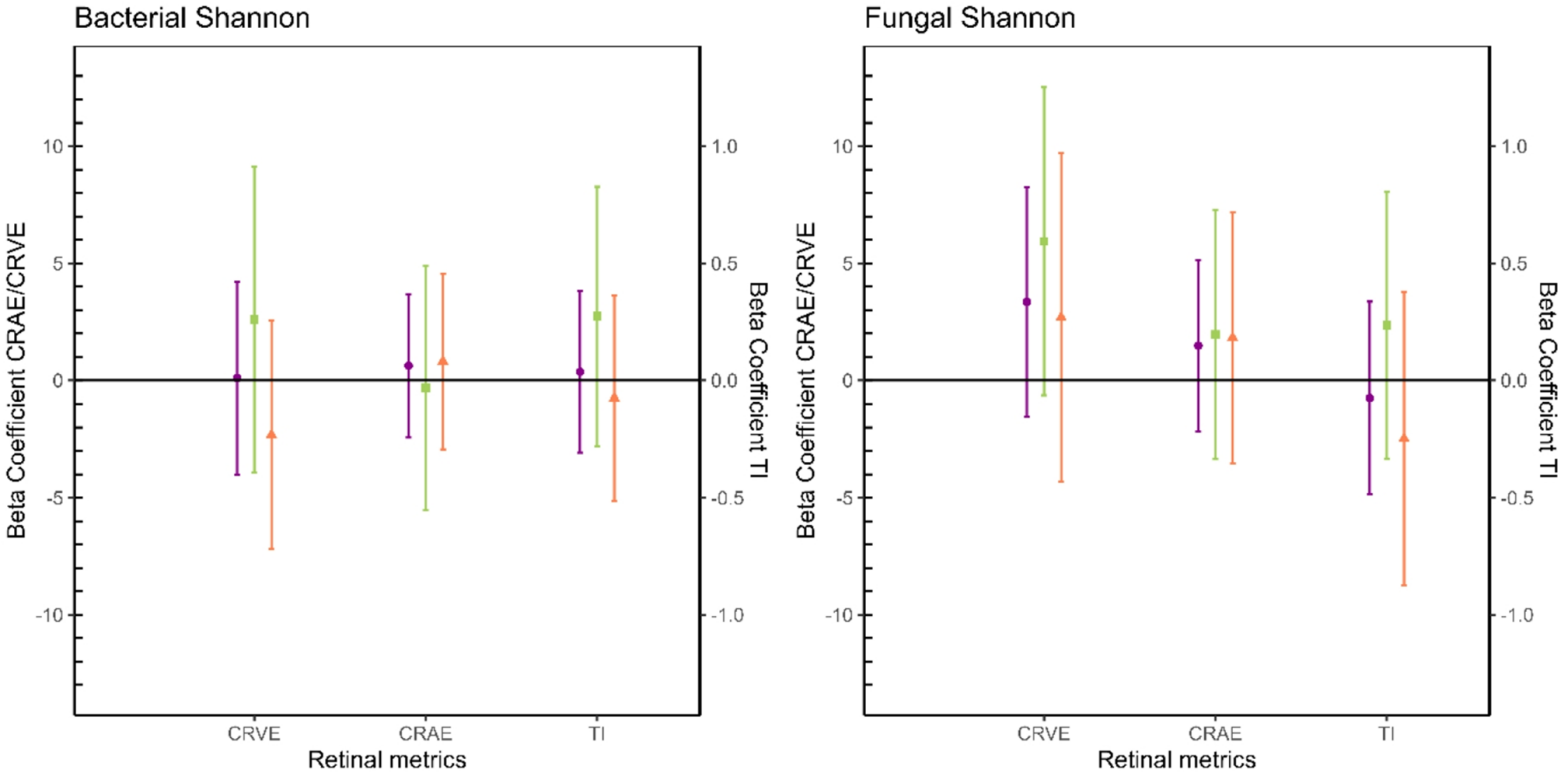
Adjusted beta coefficients and their 95% confidence intervals (CI) for Shannon indices versus retinal microcirculation outcomes in the total population (*n* = 177, purple), boys (*n* = 89, green) and girls (*n* = 88, orange). Models were adjusted for exposure counterpart (bacteria/fungi), BMI, mean arterial blood pressure, education of the mother, black carbon concentrations, proportion of high green around the residence and settled dust duration of sampling. CRVE: central retinal vein equivalent; CRAE: central retinal arterial equivalent, TI: tortuosity index.

**Fig. 4:**
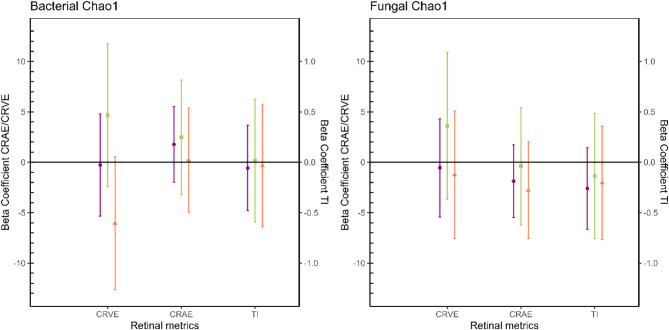
Adjusted beta coefficients and their 95% confidence intervals (CI) for Chao1 indices versus retinal microcirculation outcomes in the total population (*n* = 177, purple), boys (*n* = 89, green) and girls (*n* = 88, orange). Models were adjusted for exposure counterpart (bacteria/fungi), BMI, mean arterial blood pressure, education of the mother, black carbon concentrations, proportion of high green around the residence and duration of settled dust sampling. CRVE: central retinal vein equivalent; CRAE: central retinal arterial equivalent, TI: tortuosity index.

The Figs. 2, 3 and 4 show the adjusted beta coefficients and their 95% confidence intervals (CI) for the relation between microbial loads, Shannon diversity index and Chao1 richness index, respectively, versus retinal microcirculation outcomes in the total population and stratified for sex. The exact values for the beta coefficients and their 95% CI presented in the figures are provided in the table S2 (supplementary material). We note here our three key observations: (1) there is an inverse association between the retinal metrics and total microbial loads (both bacterial and fungal), (2) there is a direct association between the fraction Gram-positive bacterial load and the retinal metrics, and (3) these associations are different between boys and girls.

In the total population, we observed that CRAE significantly decreased with increases in the total bacterial and fungal loads (Fig. 2). In particular, we observe a 0.28 μm decrease (CI = -0.53; -0.04) in CRAE for a 10% increase in bacterial load and a 0.27 μm decrease (CI = -0.50; -0.03) in CRAE for a 10% increase in fungal load. Next to CRAE, we also found significant associations for TI. We observed a 0.44% point increase (CI = 0.06; 0.80) in TI for an IQR increase in the fraction of Gram-positive bacterial load. As visualized in Figs. 3 and 4, we haven’t observed any significant associations between fungal and bacterial richness and diversity indices and retinal microcirculation metrics. Moreover, we observed little consistency in the direction of the association between these alpha diversity indices and the outcomes. After stratifying by sex, we generally observed stronger associations among boys compared to girls, with statistically significant associations among boys for CRAE with fungal load (β=-0.42; CI = -0.82; -0.03) and for TI with bacterial load (β=-0.05; CI = -0.09; -0.0007). Notably, we observe partial differences in the direction of the associations between microbial exposures (loads, richness and diversity) and retinal microcirculation in male and female subgroups, especially pronounced for CRVE.

### Differential abundance of individual bacterial and fungal taxa in dependency of retinal microcirculation

Using ANCOM BC2 we identified individual bacterial and fungal genera from the amplicon sequencing datasets that were differentially abundant in samples grouped by outcome variables, i.e. CRAE, CRVE and TI. Similarly, random forest was used to identify which bacterial and fungal genera were most contributing to the retinal microcirculation characteristics. The median with its IQR and importance or q-value of the identified genera are presented in the supplementary material (Influential microbial genera: Tables S3 to S11). We used the identified genera to adjust our models, in an effort to assess their explanatory value. The exact beta coefficients and their 95% CI for the relation between each retinal microcirculation characteristic and the microbial loads with and without adjustment for the identified genera) are shown in Table S12 in the supplementary material. In all of the relations between the microbial exposures and retinal microcirculation characteristics, adjusting for the most influential genera did not substantially change the beta coefficients.

### Sensitivity analyses

In the sensitivity analyses, we checked the robustness of our models by assessing the impact of influential observations, replacing some confounders and determinants in the models, and assessing the impact of missing data. Regarding the influential observations, models built on the total population had a median of 8.7 influential observations (IQR = 2.5). For stratified models, the median number of influential observations was 3.8 (IQR = 1) and 4.4 (IQR = 2.5) for the girls and boys, respectively. The children in which the influential observations were made differed between the models although for each outcome, the groups were largely similar. Generally, excluding influential observations resulted in associations closer to the null but the direction of the association did not change. Only the association between the fungal load and CRAE in the total population remained statistically significant (data on influential observations not shown). The assessment of robustness by replacing some confounders and determinants in the models was done by replacing mean arterial blood pressure, BMI, black carbon concentrations and residential high green with systolic blood pressure, zBMI, NO2 concentrations and residential low green, respectively. The results did not change (data not shown). Last, we looked at the impact of missing data. There were no substantial differences in the association estimates between the complete case analysis and the models on the multiple imputed data sets (Table S13).

## Discussion

We show for the first time that the features of the indoor environmental microbiome may influence the retinal microcirculation of pre-school-aged children. We discovered that high total bacterial and fungal loads in residential dust were associated with lower retina vessel diameters and tortuosity. In contrast, high representation of Gram-positive bacteria in the total bacterial loads increased vessel diameters and tortuosity, in several cases significantly. Adjusting the models for individual bacterial and fungal genera that were associated with the outcomes did not change the studied associations, indicating that the observed effects were in fact due to overall microbial loads rather than compositional characteristics within the dust microbiota. Further supporting this notion, neither richness nor diversity of the microbiota were associated with retinal microcirculation. As expected, ignoring influential observations did impact the results, but not enough to conclude that our findings are determined by a few observations within the population. When stratifying the study population by sex, the associations were generally stronger (and/or persist) among boys compared to girls.

To the best of our knowledge, there are no previous studies that explored the associations between indoor environmental microbiome and the retinal microcirculation. However, there is an extensive body of literature that has studied the systemic mechanisms underlying the health outcomes of environmental microbial exposures, largely focusing on asthma and allergies, and in many cases carried out in the context of farm environments^7,61–63^. These mechanisms are assumed to be immune-related^2–4,64–69^. A Finnish research group showed an impact of the environmental microbiome on the immune system of both children^70^ and adults^71^ in small intervention trials. Here, we investigated the retinal microcirculation in children, a cardiovascular biomarker that has been linked to multiple health outcomes, including immunological outcomes^25–31^, to explore the potential mechanisms underlying the health impacts of exposure to the environmental microbiome.

Cognitive performance is potentially impacted by environmental microbiome exposures. In the same cohort we previously reported that an increased Gram-positive bacterial load in residential house dust was associated with worse cognitive performance, while an increased fungal Shannon index was associated with better cognitive performance^11^. In addition, Luyten et al. described that widening of the CRVE, CRAE and higher TI was associated with worse cognitive performance in children aged 4 to 6 years, although only for CRVE, the significance level of 0.05 was reached. Based on these studies, we expected that CRVE, CRAE and TI would be directly associated with the Gram-positive bacterial load and inversely with the Shannon fungal index. In our study, we observed the expected direct association between the fraction of Gram-positive bacterial load and the retinal microcirculation characteristics, but not the expected inverse association of the retinal microcirculation characteristics and the fungal Shannon index. Our results suggest that exposure to a more Gram-positive dominated bacterial load in house dust is associated with increased arterial vessel diameters and tortuosity in the retinal microcirculation. Although these changes are subtle, alterations in retinal microvascular morphology have been consistently linked to cardiovascular and neurovascular risk, underscoring their potential clinical relevance as early indicators of systemic vascular health. In addition, these microvascular changes can decrease blood flow in the brain^32,33^ and could potentially help explain the cognitive effects suggested in previous studies^11,19,36^.

We observed differences in the strength and the direction of the associations between boys and girls. It is at present unclear why boys are more responsive with their retinal microcirculation metrics upon exposure to microbes. Sex-specific differences in immune response could be one explanation^72^, but such differences are reported to be more pronounced after puberty. Alternatively, boys could be more directly exposed to the environmental microbiome due to sex-specific play habits. Research shows that boys play more outside^58,59^ and the outdoor contributes to the indoor microbiome^45^. It is plausible to consider that sex-specific play differences could lead to an increased exposure in boys. We did adjust for the proportion of residential green spaces in our models. However, we didn’t collect data on ‘hours of playing or being active outdoors’ or ‘type of play’ in this study.

We acknowledge our study limitations. Firstly, the sample size was small and it is likely that we lack statistical power to detect small effect size associations. This is especially true in the stratified analyses by sex. Nevertheless, we had enough power to show statistically significant associations with loads in the total population and the boys. In addition, the observed trends were consistent across all strata and in all our sensitivity analysis. Secondly, we conducted our study in a subsample of the ENVIRONAGE population and we had a (limited) number of missing data on the outcome measurements, which may have introduced selection bias. Regarding the first, Janssen et al.^42^ compared the main characteristics of the ENVIRONAGE birth cohort to the Flemish population and concluded that the cohort is broadly representative of the Flemish population. The characteristics of our study population did not substantially differ from those of the full cohort or the Flemish population as previously described^42^. The only notable difference was a slightly higher proportion of highly educated mothers in our study population. Regarding the missing information in the outcome, we did not observe significant differences among participants with and without information on the outcome and assumed missing completely at random. Nevertheless, to rule out possible selection bias due to missing at random, we used multiple imputation techniques. The results of both complete-case and imputed analyses were comparable. Therefore, we consider that in our study the risk of selection bias, both related to the use of a subsample and to missing data, is limited.

Thirdly, information on the child’s immune system, like cytokine levels, was not available. Given our DAG (see supplementary material Figure S1) and hypothesis, this variable is central in the relation between the environmental microbiome and its health effects (including effects on the retinal microcirculation). Having this information available would have helped us further exploring the underlying mechanisms. Lastly, the design of the data collection was cross-sectional, and it is not open question how well the indoor microbial exposure assessed at the age of 4–6 years reflects exposure earlier in childhood. Considering this, we collected airborne settled dust accumulated over a period of several weeks in the homes’ living rooms, a sample type that represents a longer term integrated exposure and avoiding the known temporal and spatial variability of short-term (air) samples that poorly reflect human exposure indoors over time^73^. Moreover, a study in Spain concluded that, although there are climatic and seasonal variations, the urban atmospheric microbiome seems relatively stable over a 2-year period^74^. In our study, microbiome sampling was performed in spring, ignoring the other seasons but likely providing a comparable and representable sample for the child’s long-term exposure. The same is true for the retinal microcirculation^34,75^. Yearly sampling points for both exposure and outcome could paint a clearer picture.

Notwithstanding the aforementioned limitations, this study has multiple strengths. Firstly, state-of-the art measurement methods and analysis were used to calculate microbial diversity, load, and composition and retinal microcirculation characteristics. Secondly, the ENVIRONAGE is an exceptionally well-characterized cohort – considering both exposure and health variables - and accurate measurements of all known confounding variables were available, allowing for adjustment during modelling. The potential for residual confounding can never truly be eliminated, and perhaps data on the immune system, gut microbiome and indoor air quality would allow for even better model adjustment. But all of these are linked to already included variables and adding them could cause over-adjusting. Lastly, all variables (except for maternal education) were objective measures, making recall bias unlikely. We constructed robust models that handled variable variations, multiple imputation and influential observations. These models succeeded in offering new and robust information on the effects of exposure to the environmental microbiome. These strengths give us confidence in our conclusion.

There is barely any research published on health effects other than allergies and asthma, associated with early life exposure to the residential microbiome. We are the first to investigate the relationship between the retinal microcirculation and the indoor, house dust microbiota. Our findings imply that the properties of retinal microcirculation in children between the ages of 4 and 6 may be influenced by the indoor environmental microbiome, specifically the quantity of microorganisms present, and that this may differ for boys and girls. Our results suggest that a higher proportion of Gram-positive bacteria in the total bacterial load in house dust samples is associated with retinal microvascular changes that are frequently linked to adverse health outcomes^19,25–40^ while exposure to higher total bacterial loads and higher fungal loads could be beneficial.

While the associations of the environmental microbiome with retinal vessel metrics may be weaker compared to other determinants of the retinal microcirculation (e.g. age or cardiovascular risk factors), they remain relevant. The environmental microbiome represents a potentially modifiable factor, and its exposure is not limited to high-risk populations but is population-wide. Evidence from other studies shows that early-life microbial exposures can protect against asthma and allergies and could influence cognitive development^7,11,19,36,61–63^. In this study, we add evidence of a potential impact on the development of the cardiovascular system. Consequently, our findings may provide new insights into the effects of environmental microbiota by elucidating the mechanism by which exposure to microbes indoor could be associated with cardiovascular and neurological health outcomes. It is too early to make specific recommendations on ideal indoor microbiota characteristics during the first years of life. However, our study supports the growing body of evidence that the environmental microbiota and absence of crucial stimuli thereof could cause adverse developmental health outcomes that go beyond asthma and allergies.

## Supplementary Information

Below is the link to the electronic supplementary material.

Supplementary Material 1

## Data Availability

Bacterial and fungal amplicon sequencing data are available under ENA (European Nucleotide Archive) at EMBL-EBI under accession number PRJEB89387 ( https:/www.ebi.ac.uk/ena/browser/view/PRJEB89387 ).
