# Supplementary material for: The association between preschoolers’ retinal microcirculation and the indoor microbial environment: results of the ENVIRONAGE birth cohort
Source: Sci Rep. 2025 Oct 6;15:34718. doi: 10.1038/s41598-025-18379-y (PMC12500906; doi:10.1038/s41598-025-18379-y)
Supplement: Supplementary file 1 — Supplementary Material 1 [file 41598_2025_18379_MOESM1_ESM.docx]

The association between preschoolers’ retinal microcirculation and the indoor microbial environment: results of the ENVIR*ON*AGE birth cohort.

Wouter van Dyck¹, Martin Täubel², Pauli Tuoresmäki², Yinthe Dockx³, Leen Luyten³, Leen Rasking³, Patrick De Boever³^,^⁴, Tim S. Nawrot³^,^⁵ and Lidia Casas¹^,^⁶^,^⁷

**Affiliations:**

¹ Social Epidemiology and Health Policy (SEHPO), Department of Family Medicine and Population Health, University of Antwerp, Universiteitsplein 1, 2610 Wilrijk, Belgium.

² Finnish Institute for Health and Welfare, Environmental Health Team, Neulaniementie 4, 70701 Kuopio, Finland.

³ Centre for Environmental Sciences, Hasselt University, Agoralaan, 3590 Diepenbeek, Belgium.

⁴ Antwerp University Hospital (UZA), Drie Eikenstraat 655, 2650 Edegem, Belgium.

⁵ Center for Environment and Health, Department of Public Health, KU Leuven, Herestraat 49, 3000 Leuven, Belgium.

⁶ Institute for Environment and Sustainable Development (IMDO), University of Antwerp, Universiteitsplein 1, 2610 Wilrijk, Belgium.

⁷ Laboratory of Applied Microbiology and Biotechnology (LAMB), Department of Bioscience Engineering, University of Antwerp, Groenenborgerlaan 171, 2020 Antwerp, Belgium.

**Corresponding Author:**

Lidia Casas (lidia.casas@uantwerpen.be)

Supplementary materials

Figure S1: The directed acyclic graph (DAG) that depicts our assumptions on relations between our exposure and outcome of interest.
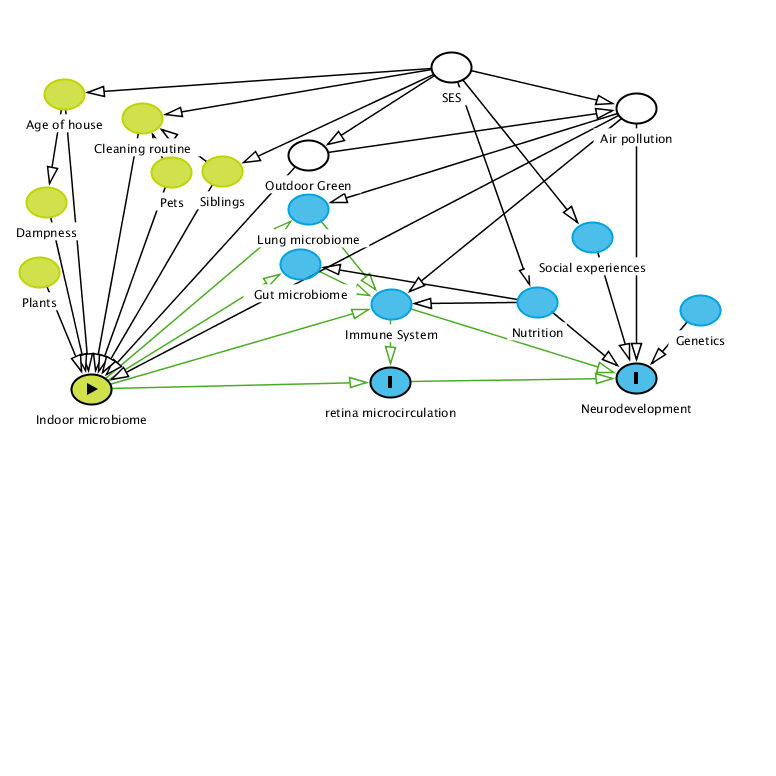


Table S1. Characteristics of the population with (n=160) and without (n=17) information on the retinal microcirculation characteristics. None of the comparisons was statistically significant (p-values>0.05).

|  |  |  | Not missing (n=160) | Missing (n=17) |
| --- | --- | --- | --- | --- |
| **Child characteristics** | | |  |  |
|  | Female (n and %) | | 80 (50%) | 8 (47.1%) |
|  | Age (years) | | 4.4 (0.4) | 4.3 (0.2) |
|  | BMI (kg/m²) | | 15.9 (1.6) | 16 (0.8) |
| **Maternal characteristics** | | |  |  |
|  | Age (years) | | 30 (5) | 31 (4) |
|  | High education (n and %) | | 110 (68.8%) | 13 (76.5%) |
| **Residential microbial environment** | | |  |  |
|  | Bacteria in settled dust | |  |  |
|  |  | Shannon index | 7.1 (1.1) | 7.4 (0.8) |
|  |  | Chao1 index | 404.9 (210.9) | 471.8 (164.7) |
|  |  | Gram positive loads (CE/m2/day) | 158788 (196682) | 146783 (141964) |
|  |  | Gram negative loads (CE/m2/day) | 288886 (339661) | 203993 (336982) |
|  | Fungi in settled dust | |  |  |
|  |  | Shannon index | 3.4 (1.4) | 3.2 (0.9) |
|  |  | Chao1 index | 132.8 (82.8) | 109.6 (55.1) |
|  |  | Load (CE/m2/day) | 33724 (42648) | 48290 (81868) |

Figure S2. Correlation matrix of microbial exposures and retinal microcirculation characteristics among girls (A) and boys (B). The size of the circle indicates the strength of the correlation. The color of the circle indicates the direction of the correlation with blue being directly correlated and red inversely.


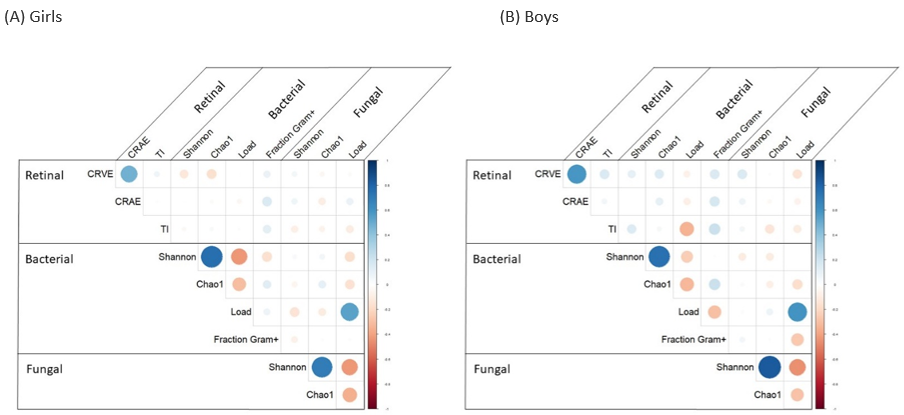


CRVE: central retinal vein equivalent; CRAE: central retinal arterial equivalent, TI: tortuosity index

Figure S3. Correlation matrix of household and retinal microcirculation characteristics in the complete population (A), among girls (B) and among boys (C). The size of the circle indicates the strength of the correlation. The color of the circle indicates the direction of the correlation with blue being directly correlated and red inversely.


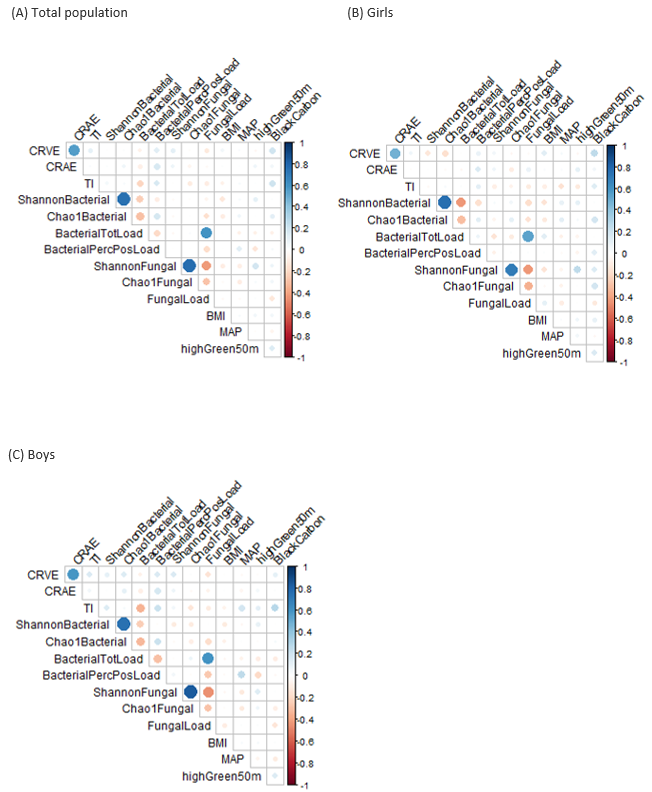


CRVE: central retinal vein equivalent; CRAE: central retinal arterial equivalent, TI: tortuosity index, BMI: Body mass index, MAP: Mean arterial blood pressure.

Table S2. Adjusted associations (beta coefficient and 95% confidence interval (CI)) for each retinal microcirculation characteristic versus each microbiome characteristic, obtained by linear regression models with adjustment for socio-economic status (by proxy of maternal education), air pollution (by proxy of black carbon concentrations), proportion of residential high green within 50m of the household, child mean arterial blood pressure and zBMI, and duration of sampling.

|  |  |  | Total (n=160) | Girls (n=80) | Boys (n=80) |
| --- | --- | --- | --- | --- | --- |
|  |  |  | beta (95% CI) | beta (95% CI) | beta (95% CI) |
| CRVE | |  |  |  |  |
|  | Bacteria | |  |  |  |
|  |  | Shannon | 0.10 (-4.02 ; 4.22) | -2.31 (-7.19 ; 2.56) | 2.60 (-3.93 ; 9.13) |
|  |  | Chao1 | -0.28 (-5.35 ; 4.79) | -6.05 (-12.66 ; 0.56) | 4.66 (-2.43 ; 11.75) |
|  |  | Load | -0.22 (-0.55 ; 0.12) | -0.15 (-0.56 ; 0.26) | -0.20 (-0.75 ; 0.35) |
|  |  | Gram+ (%) | 2.81 (-1.68 ; 7.30) | -1.84 (-8.09 ; 4.41) | 4.10 (-2.22 ; 10.43) |
|  | Fungi | |  |  |  |
|  |  | Shannon | 3.35 (-1.54 ; 8.24) | 2.69 (-4.32 ; 9.70) | 5.94 (-0.64 ; 12.52) |
|  |  | Chao1 | -0.54 (-5.41 ; 4.33) | -1.25 (-7.57 ; 5.07) | 3.61 (-3.66 ; 10.88) |
|  |  | load | -0.24 (-0.56 ; 0.08) | -0.21 (-0.61 ; 0.18) | -0.29 (-0.79 ; 0.22) |
| CRAE | |  |  |  |  |
|  | Bacteria | |  |  |  |
|  |  | Shannon | 0.62 (-2.43 ; 3.68) | 0.81 (-2.93 ; 4.56) | -0.32 (-5.52 ; 4.89) |
|  |  | Chao1 | 1.77 (-1.98 ; 5.53) | 0.20 (-4.97 ; 5.37) | 2.49 (-3.18 ; 8.15) |
|  |  | Load | -0.28 (-0.53 ; -0.04) | -0.16 (-0.47 ; 0.16) | -0.41 (-0.84 ; 0.02) |
|  |  | Gram+ (%) | 3.20 (-0.11 ; 6.52) | 1.51 (-3.26 ; 6.28) | 4.18 (-0.80 ; 9.17) |
|  | Fungi | |  |  |  |
|  |  | Shannon | 1.48 (-2.17 ; 5.13) | 1.83 (-3.53 ; 7.18) | 1.96 (-3.36 ; 7.27) |
|  |  | Chao1 | -1.87 (-5.48 ; 1.73) | -2.78 (-7.57 ; 2.01) | -0.38 (-6.19 ; 5.43) |
|  |  | load | -0.27 (-0.50 ; -0.03) | -0.15 (-0.45 ; 0.15) | -0.42 (-0.82 ; -0.03) |
| TII |  |  |  |  |  |
|  | Bacteria | |  |  |  |
|  |  | Shannon | 0.04 (-0.31 ; 0.38) | -0.07 (-0.51 ; 0.36) | 0.27 (-0.28 ; 0.83) |
|  |  | Chao1 | -0.06 (-0.48 ; 0.37) | -0.03 (-0.64 ; 0.57) | 0.02 (-0.59 ; 0.62) |
|  |  | Load | -0.02 (-0.05 ; 0.01) | 0.00 (-0.04 ; 0.04) | -0.05 (-0.09 ; 0.00) |
|  |  | Gram+ (%) | 0.44 (0.06 ; 0.81) | 0.26 (-0.29 ; 0.82) | 0.52 (-0.01 ; 1.05) |
|  | Fungi | |  |  |  |
|  |  | Shannon | -0.07 (-0.49 ; 0.34) | -0.25 (-0.87 ; 0.38) | 0.24 (-0.33 ; 0.80) |
|  |  | Chao1 | -0.26 (-0.66 ; 0.14) | -0.20 (-0.77 ; 0.36) | -0.14 (-0.76 ; 0.48) |
|  |  | load | -0.02 (-0.05 ; 0.01) | -0.01 (-0.05 ; 0.02) | -0.03 (-0.08 ; 0.01) |

CRVE: central retinal vein equivalent; CRAE: central retinal arterial equivalent, TI: tortuosity index

Table S3. Mean relative abundance with its interquartile range and the importance (Imp.) for the top 5 influential bacterial genera that were identified by Random Forest in relation to CRAE in the total data set (n=177). Random Forest was performed on the relative abundance data of all bacterial genera.

| **Phylum** | **Class** | **Order** | **Family** | **Genus** | **Mean (IQR)** | **Imp.** |
| --- | --- | --- | --- | --- | --- | --- |
| Firmicutes | Bacilli | Lactobacillales | Carnobacteriaceae | Jeotgalibaca | 1.30e-04 (5.10e-04) | 965 |
| Proteobacteria | Deltaproteobacteria | Desulfuromonadales | Geobacteraceae | Geobacter | 4.31e-05 (1.56e-04) | 643 |
| Cyanobacteria | Oxyphotobacteria | Oxyphotobacteria_Incertae_Sedis | Unknown_Family | Leptolyngbya_ANT.L52.2 | 6.85e-05 (2.41e-04) | 584 |
| Firmicutes | Clostridia | Clostridiales | Clostridiaceae_1 | Clostridium_sensu_stricto_1 | 1.06e-02 (1.00e-02) | 518 |
| Firmicutes | Bacilli | Lactobacillales | Leuconostocaceae | Leuconostoc | 1.64e-03 (5.87e-03) | 437 |

Table S4. Mean relative abundance with its interquartile range and q-value (Q) for the influential bacterial genera that had a q value below 1 using the ANCOM BC2 method in relation to CRAE on the total data set (n=177). ANCOM BC2 was performed on the absolute counts data of all bacterial genera.

| **Phylum** | **Class** | **Order** | **Family** | **Genus** | **Mean (IQR)** | **Q** |
| --- | --- | --- | --- | --- | --- | --- |
| Firmicutes | Bacilli | Lactobacillales | Carnobacteriaceae | Jeotgalibaca | 1.30e-04 (5.10e-04) | 0.75 |

Table S5. Mean relative abundance with its interquartile range and importance (Imp.) for the top 5 influential bacterial genera that were identified by Random Forest in relation to CRVE in the total data set (n=177). Random Forest ran on the relative abundance data of all bacterial genera.

| **Phylum** | **Class** | **Order** | **Family** | **Genus** | **Mean (IQR)** | **Imp.** |
| --- | --- | --- | --- | --- | --- | --- |
| Firmicutes | Clostridia | Clostridiales | Ruminococcaceae | Subdoligranulum | 8.81e-04 (1.35e-03) | 2148 |
| Proteobacteria | Alphaproteobacteria | Rhodobacterales | Rhodobacteraceae | Rubellimicrobium | 8.90e-03 (7.66e-03) | 1387 |
| Proteobacteria | Alphaproteobacteria | Sphingomonadales | Sphingomonadaceae | Sphingopyxis | 1.34e-04 (3.16e-04) | 1161 |
| Proteobacteria | Alphaproteobacteria | Elsterales | NA | NA | 1.94e-04 (4.65e-04) | 860 |
| Proteobacteria | Gammaproteobacteria | Betaproteobacteriales | Burkholderiaceae | Lautropia | 1.49e-03 (1.43e-03) | 719 |

NA: identification of phylum, class, order, family or genus was not possible based on the sequencing results

Table S6. Mean relative abundance with its interquartile range and q-value (Q) for the influential bacterial genera that had a q value below 1 using the ANCOM BC2 method in relation to CRVE on the total data set (n=177). ANCOM BC2 ran on the absolute counts data of all bacterial genera.

| **Phylum** | **Class** | **Order** | **Family** | **Genus** | **Mean (IQR)** | **Q** |
| --- | --- | --- | --- | --- | --- | --- |
| Proteobacteria | Alphaproteobacteria | Sphingomonadales | Sphingomonadaceae | Sphingopyxis | 1.34e-04 (3.16e-04) | 0.00153 |

Table S7. Mean relative abundance with its interquartile range and importance (Imp.)for the top 5 influential bacterial genera that were identified by Random Forest in relation to TI in the total data set (n=177). Random Forest ran on the relative abundance data of all bacterial genera.

| **Phylum** | **Class** | **Order** | **Family** | **Genus** | **Mean (IQR)** | **Imp.** |
| --- | --- | --- | --- | --- | --- | --- |
| Actinobacteria | Actinobacteria | Actinomycetales | Actinomycetaceae | Actinomyces | 2.39e-03 (2.62e-03) | 10 |
| Firmicutes | Clostridia | Clostridiales | Lachnospiraceae | Catonella | 1.15e-05 (6.30e-05) | 9 |
| Cyanobacteria | Oxyphotobacteria | Nostocales | Coleofasciculaceae | Microcoleus_PCC-7113 | 6.93e-05 (2.57e-04) | 7 |
| Bacteroidetes | Bacteroidia | Bacteroidales | Prevotellaceae | Prevotella_7 | 8.72e-04 (1.39e-03) | 6 |
| Firmicutes | Negativicutes | Selenomonadales | Veillonellaceae | Veillonella | 1.67e-03 (2.16e-03) | 6 |

Table S8. Mean relative abundance with its interquartile range and importance (Imp.) for the top 5 influential fungal genera that were identified by Random Forest in relation to CRAE in the total data set (n=177). Random Forest ran on the relative abundance data of all fungal genera.

| **Phylum** | **Class** | **Order** | **Family** | **Genus** | **Mean (IQR)** | **Imp.** |
| --- | --- | --- | --- | --- | --- | --- |
| Ascomycota | Dothideomycetes | Capnodiales | Capnodiales_fam_Incertae_sedis | Phaeotheca | 7.51e-05 (2.31e-04) | 1104 |
| Ascomycota | Eurotiomycetes | Eurotiales | Aspergillaceae | Penicillium | 2.89e-02 (5.51e-02) | 1085 |
| Ascomycota | Dothideomycetes | Pleosporales | Phaeosphaeriaceae | Phaeosphaeria | 1.25e-03 (1.59e-03) | 981 |
| Ascomycota | Lecanoromycetes | Teloschistales | Teloschistaceae | Xanthocarpia | 9.89e-06 (9.15e-05) | 752 |
| Ascomycota | Dothideomycetes | Pleosporales | Cucurbitariaceae | Pyrenochaetopsis | 5.21e-04 (9.14e-04) | 615 |

Table S9. Mean relative abundance with its interquartile range and importance (Imp.) for the top 5 influential fungal genera that were identified by Random Forest in relation to CRVE in the total data set (n=177). Random Forest ran on the relative abundance data of all fungal genera.

| **Phylum** | **Class** | **Order** | **Family** | **Genus** | **Mean (IQR)** | **Imp.** |
| --- | --- | --- | --- | --- | --- | --- |
| Ascomycota | Sordariomycetes | Hypocreales | Nectriaceae | Cosmospora | 7.84e-06 (7.79e-05) | 3546 |
| Ascomycota | Leotiomycetes | Helotiales | Helotiales_fam_Incertae_sedis | Xenopolyscytalum | 8.04e-06 (5.77e-05) | 1810 |
| Basidiomycota | Microbotryomycetes | Sporidiobolales | Sporidiobolaceae | Sporobolomyces | 2.02e-02 (2.00e-02) | 1383 |
| Ascomycota | Sordariomycetes | Xylariales | Diatrypaceae | Eutypa | 1.17e-04 (3.45e-04) | 1332 |
| Ascomycota | Dothideomycetes | Capnodiales | Mycosphaerellaceae | Mycosphaerella | 4.08e-01 (1.18e-01) | 1275 |

Table S10. Mean relative abundance with its interquartile range and importance (Imp.) for the top 5 influential fungal genera that were identified by Random Forest in relation to TI in the total data set (n=177). Random Forest ran on the relative abundance data of all fungal genera.

| **Phylum** | **Class** | **Order** | **Family** | **Genus** | **Mean (IQR)** | **Imp.** |
| --- | --- | --- | --- | --- | --- | --- |
| Basidiomycota | Agaricomycetes | Polyporales | Meruliaceae | Bjerkandera | 1.39e-03 (3.37e-03) | 17 |
| Ascomycota | Dothideomycetes | Capnodiales | Cladosporiaceae | Cladosporium | 8.40e-02 (3.30e-02) | 13 |
| Ascomycota | Sordariomycetes | Hypocreales | Hypocreaceae | Trichoderma | 4.17e-04 (1.69e-03) | 10 |
| Ascomycota | Dothideomycetes | Pleosporales | Pleosporaceae | Alternaria | 1.18e-02 (7.71e-03) | 9 |
| Basidiomycota | Agaricomycetes | Agaricales | NA | NA | 1.55e-04 (4.36e-04) | 9 |

NA: identification of phylum, class, order, family or genus was not possible based on the sequencing results

Table S11. The mean relative abundance with its interquartile range and q-value (Q) for the influential fungal genera that had a q value below 1 using the ANCOM BC2 method in relation to TI on the total data set (n=177). ANCOM BC2 ran on the absolute counts data of all fungal genera.

| **Phylum** | **Class** | **Order** | **Family** | **Genus** | **Mean (IQR)** | **Q** |
| --- | --- | --- | --- | --- | --- | --- |
| Basidiomycota | Agaricomycetes | Polyporales | Meruliaceae | Bjerkandera | 1.39e-03 (3.37e-03) | 0,62 |
| Basidiomycota | Agaricomycetes | Russulales | NA | NA | 3.19e-04 (1.11e-03) | 0,73 |
| Basidiomycota | Agaricomycetes | Russulales | Stereaceae | Stereum | 8.08e-04 (1.62e-03) | 0,90 |

NA: identification of phylum, class, order, family or genus was not possible based on the sequencing results

Table S12. Adjusted associations (beta coefficient and 95% confidence interval (CI)) for each retinal microcirculation characteristic versus the microbial loads as reported in table S1 (Main), adjusted for influential genera as identified by Random Forest (RF), and adjusted for influential genera as identified by ANCOM BC2. All models are adjusted for socio-economic status (by proxy of maternal education), air pollution (by proxy of black carbon concentrations), proportion of residential high green within 50m of the household, child mean arterial blood pressure and zBMI, and duration of sampling.

|  | | **Beta (95%CI)  Main** | **Beta (95%CI) RF** | **Beta (95%CI) ANCOM BC2** |
| --- | --- | --- | --- | --- |
| **CRAE** | | | | |
|  | Bacterial loads | -0.28 (-0.53 ; -0.036) | -0.19 (-0.42 ; 0.046) | -0.25 (-0.49 ; -0.015) |
|  | Gram+ (%) | 3.2 (-0.11 ; 6.5) | 2.4 (-0.73 ; 5.6) | 2.7 (-0.43 ; 5.9) |
|  | Fungal loads | -0.27 (-0.5 ; -0.03) | -0.22 (-0.45 ; 0.02) | NA |
| **CRVE** | | | | |
|  | Bacterial loads | -0.22 (-0.55 ; 0.12) | -0.11 (-0.43 ; 0.21) | -0.27 (-0.59 ; 0.051) |
|  | Gram+ (%) | 2.8 (-1.7 ; 7.3) | 2.5 ( -2 ; 7) | NA |
|  | Fungal loads | -0.24 (-0.56 ; 0.084) | -0.2 (-0.52 ; 0.11) | NA |
| **TI** | | | | |
|  | Bacterial loads | -0.018 (-0.046 ; 0.0097) | -0.01 (-0.038 ; 0.018) | NA |
|  | Gram+ (%) | 0.44 (0.065 ; 0.81) | 0.59 (0.22 ; 0.97) | NA |
|  | Fungal loads | -0.021 (-0.048 ; 0.0052) | -0.033 (-0.061 ; -0.0039) | -0.031 (-0.057 ; -0.0053) |

NA: No influential genera identified by ANCOM BC2

Table S13. Adjusted associations (beta coefficient and 95% confidence interval (CI)) for each retinal microcirculation characteristic versus each microbiome characteristic on the imputed dataset (n=177). The association estimates are adjusted for socio-economic status (by proxy of maternal education), air pollution (by proxy of black carbon concentrations), proportion of residential high green within 50m of the household, child mean arterial blood pressure and zBMI, and duration of sampling.

|  |  |  | Total (n=177) | Girls (n=88) | Boys (n=89) |
| --- | --- | --- | --- | --- | --- |
|  |  |  | beta (95% CI) | beta (95% CI) | beta (95% CI) |
| CRVE |  |  |  |  |  |
|  | Bacteria |  |  |  |  |
|  |  | Shannon | 0.05 (-4.05 : 4.16) | -2.23 (-7.15 : 2.69) | 2.85 (-3.64 : 9.34) |
|  |  | Chao1 | -0.24 (-5.30 : 4.83) | -5.82 (-12.47 : 0.83) | 5.06 (-2.04 : 12.16) |
|  |  | Gram positive loads | -0.09 (-0.40 : 0.22) | -0.19 (-0.55 : 0.16) | 0.02 (-0.54 : 0.59) |
|  |  | Gram negative loads | -0.25 (-0.58 : 0.08) | -0.16 (-0.59 : 0.26) | -0.26 (-0.77 : 0.26) |
|  | Fungi |  |  |  |  |
|  |  | Shannon | 3.55 (-1.33 : 8.42) | 2.65 (-4.46 : 9.75) | 6.09 (-0.42 : 12.60) |
|  |  | Chao1 | -0.30 (-5.17 : 4.56) | -1.37 (-7.83 : 5.10) | 3.53 (-3.75 : 10.80) |
|  |  | Loads | -0.24 (-0.56 : 0.08) | -0.21 (-0.61 : 0.19) | -0.33 (-0.83 : 0.18) |
| CRAE |  |  |  |  |  |
|  | Bacteria |  |  |  |  |
|  |  | Shannon | 0.58 (-2.48 : 3.64) | 0.85 (-2.92 : 4.62) | -0.26 (-5.50 : 4.97) |
|  |  | Chao1 | 1.71 (-2.06 : 5.48) | 0.28 (-4.90 : 5.47) | 2.36 (-3.39 : 8.11) |
|  |  | Gram positive loads | -0.16 (-0.39 : 0.07) | -0.12 (-0.39 : 0.16) | -0.25 (-0.70 : 0.20) |
|  |  | Gram negative loads | -0.32 (-0.56 : -0.07) | -0.18 (-0.51 : 0.14) | -0.42 (-0.82 : -0.01) |
|  | Fungi |  |  |  |  |
|  |  | Shannon | 1.56 (-2.10 : 5.21) | 1.86 (-3.57 : 7.28) | 2.07 (-3.26 : 7.40) |
|  |  | Chao1 | -1.76 (-5.38 : 1.86) | -2.76 (-7.65 : 2.14) | -0.12 (-6.00 : 5.75) |
|  |  | Loads | -0.27 (-0.50 : -0.03) | -0.15 (-0.45 : 0.15) | -0.43 (-0.83 : -0.04) |
| TI |  |  |  |  |  |
|  | Bacteria |  |  |  |  |
|  |  | Shannon | 0.03 (-0.31 : 0.38) | -0.07 (-0.51 : 0.37) | 0.25 (-0.30 : 0.81) |
|  |  | Chao1 | -0.07 (-0.50 : 0.35) | -0.05 (-0.65 : 0.56) | -0.02 (-0.64 : 0.60) |
|  |  | Gram positive loads | -0.01 (-0.03 : 0.02) | 0.00 (-0.03 : 0.03) | -0.02 (-0.07 : 0.03) |
|  |  | Gram negative loads | -0.02 (-0.05 : 0.00) | 0.00 (-0.04 : 0.03) | -0.05 (-0.09 : 0.00) |
|  | Fungi |  |  |  |  |
|  |  | Shannon | -0.08 (-0.49 : 0.33) | -0.28 (-0.91 : 0.35) | 0.21 (-0.36 : 0.78) |
|  |  | Chao1 | -0.26 (-0.67 : 0.15) | -0.25 (-0.82 : 0.33) | -0.12 (-0.74 : 0.51) |
|  |  | Loads | -0.02 (-0.05 : 0.01) | -0.01 (-0.05 : 0.02) | -0.03 (-0.08 : 0.01) |

CRVE: central retinal vein equivalent; CRAE: central retinal arterial equivalent, TI: tortuosity index
